# The Homeostasis of *Plasmodium falciparum*-Infected Red Blood Cells

**DOI:** 10.1371/journal.pcbi.1000339

**Published:** 2009-04-03

**Authors:** Jakob M. A. Mauritz, Alessandro Esposito, Hagai Ginsburg, Clemens F. Kaminski, Teresa Tiffert, Virgilio L. Lew

**Affiliations:** 1 Department of Chemical Engineering and Biotechnology, University of Cambridge, Cambridge, United Kingdom; 2 Department of Physiology, Development and Neuroscience, University of Cambridge, Cambridge, United Kingdom; 3 Department of Biological Chemistry, Institute of Life Sciences, The Hebrew University, Jerusalem, Israel; 4 School of Advanced Optical Technologies, Max-Planck-Research Group, Division III, University of Erlangen-Nuremberg, Erlangen, Germany; Lilly Singapore Centre for Drug Discovery, Singapore

## Abstract

The asexual reproduction cycle of *Plasmodium falciparum*, the parasite responsible for severe malaria, occurs within red blood cells. A merozoite invades a red cell in the circulation, develops and multiplies, and after about 48 hours ruptures the host cell, releasing 15–32 merozoites ready to invade new red blood cells. During this cycle, the parasite increases the host cell permeability so much that when similar permeabilization was simulated on uninfected red cells, lysis occurred before ∼48 h. So how could infected cells, with a growing parasite inside, prevent lysis before the parasite has completed its developmental cycle? A mathematical model of the homeostasis of infected red cells suggested that it is the wasteful consumption of host cell hemoglobin that prevents early lysis by the progressive reduction in the colloid-osmotic pressure within the host (the *colloid-osmoti*c hypothesis). However, two critical model predictions, that infected cells would swell to near prelytic sphericity and that the hemoglobin concentration would become progressively reduced, remained controversial. In this paper, we are able for the first time to correlate model predictions with recent experimental data in the literature and explore the fine details of the homeostasis of infected red blood cells during five model-defined periods of parasite development. The conclusions suggest that infected red cells do reach proximity to lytic rupture regardless of their actual volume, thus requiring a progressive reduction in their hemoglobin concentration to prevent premature lysis.

## Author Summary

The parasite *Plasmodium falciparum* is responsible for severe malaria in humans. The 48 hour asexual reproduction cycle of the parasite within red blood cells is responsible for the symptoms in this disease. Within this period, the parasite causes massive changes in the host red cell, increasing some metabolic activities hundredfold, making it leaky to many nutrients and waste products, and consuming most of the cell's hemoglobin, far more than it needs for its own metabolism. The challenge that we faced was to explain how the infected cell maintained its integrity throughout such a violent cycle. Seeking clues, we developed a mathematical model of an infected cell in which we encoded our current knowledge and understanding of the complex processes that control cell homeostasis. We present here for the first time a detailed description of the model and a critical analysis of its predictions in relation to the available experimental evidence. The results support the view that host-cell integrity is maintained by the progressive reduction in the hemoglobin concentration within the host cell, resulting in a reduced rate and extent of swelling.

## Introduction

*Plasmodium falciparum*, *Pf*, is responsible for the most severe form of malaria in humans, representing a major cause of morbidity and mortality, especially among children. The pathology of malaria is caused by the intraerythrocytic stage of the parasite cycle. Invasion of a red blood cell (RBC) by a *Pf* merozoite converts a metabolically languid, hemoglobin-filled cell lacking intracellular organelles and structures, into a complex double cell, with a eukaryotic organism growing and multiplying inside, protected from immune attack. After a relatively quiescent period of about 15–20 h post-invasion, infected RBCs exhibit large increases in metabolic activity and solute traffic [1]–[7] across their membrane. The elevated metabolic rate persists until late stages of development and relaxes only during the latest hours of the parasite's 48 h asexual reproduction cycle. Staines et al. [8] showed that if uninfected human RBCs were permeabilized to the same extent the uninfected cells would hemolyze by the unbalanced net gain of NaCl and osmotic water over a shorter time-course than that needed for parasite maturation and exit. How, then, is the integrity of parasitized cells preserved for the duration of the intraerythrocytic cycle, considering that they have a parasite growing to a substantial volume inside?

This puzzle prompted an investigation on how premature lysis is prevented in falciparum-infected RBCs. A mathematical model of the homeostasis of parasitized RBCs was formulated to attempt an understanding of the processes involved [9],[10]. The model encoded all known kinetic parameters relevant to the control of host red cell volume, i.e., pH, membrane potential, ion content, ion transport across the RBC membrane and parasite growth. The initial simulations with the model produced a result which led to the formulation of a “colloid-osmotic hypothesis” to explain how infected RBCs (IRBCs) resist premature lysis. The hypothesis linked lysis resistance to hemoglobin consumption, a link hitherto never suspected.

It had been well established that during the process of growth and maturation within RBCs malaria parasites ingest and digest hemoglobin (Hb) to levels far above those required by parasite protein synthesis [11]. Moreover, the amino acids produced in vast excess by hemoglobin proteolysis are rapidly released to the medium across the host RBC membrane through the so called “new permeation pathways” (NPPs) [7], [11]–[14], without apparent generation of any osmotic stress. Hb ingestion and digestion and heme detoxification, required to prevent damage to both parasite and host cell, are high energy-consuming processes [15]–[17]. It was therefore puzzling why Hb was consumed in such vast excess. The original model simulations [9] suggested that excess Hb digestion was necessary to reduce the colloid-osmotic pressure within the host cell, thus preventing its premature swelling to the critical hemolytic volume (CHV).

The simulations led to two critical predictions: (i) that excess Hb ingestion and digestion would cause not only a dramatic fall in the Hb content of the host cell, as had been established already from experimental evidence, but also a progressive and large decline in its Hb *concentration*, and (ii) that parasite volume growth together with host cell swelling late in the cell cycle would bring IRBC volumes very near their CHV. Allen and Kirk [18] argued that parasite volume growth was overestimated in the model because of its assumption that the parasite retains all of the volume taken up via the endocytotic feeding process, leading to exaggerated IRBC volume estimates. They stressed that most of the host volume ingested may be lost by different processes (e.g, Hb breakdown, Na^+^ extrusion from the parasite compartment) thus freeing up space for the parasite to expand into the host cell and limit the extent of swelling undergone by the infected cell as the parasite volume increases and cations enter the cell through colloid-osmosis. If IRBC volumes do not approach the presumed CHVs, then both the role attributed by the model to excess Hb consumption and the need for freeing space appear irrelevant. It became clear from these considerations that a thorough re-evaluation of the model assumptions and predictions in the light of past and recent experimental evidence became necessary.

In this paper we present a new, detailed analysis of the homeostasis of *P. falciparum*-infected RBCs. A full description of the model equations is given (see Text S1), and a comprehensive analysis of model assumptions and predictions over the full range of parameter values supported by experimental evidence is provided. We conclude with a re-evaluation of the colloid-osmotic hypothesis. The original model simulations [9] were generated using a restricted set of parameter values and were reported using a minimal subset of model variables, leaving out much potentially useful information on the homeostasis of IRBCs. The present account overcomes these shortcomings and defines our current understanding of the homeostasis of *P. falciparum*-infected RBCs.

## Results

The presentation of the results below, and their analysis, was guided by two specific aims: to provide a critical overview of the experimental evidence on which the information encoded in the model is based, and to allow a rational exploration of the model predictions over a wide range of possible parameter values. This exercise is necessary to enable predictions to be confronted with available results in the literature, to outline the open questions in the field and to direct future research.

**Figure 1:**
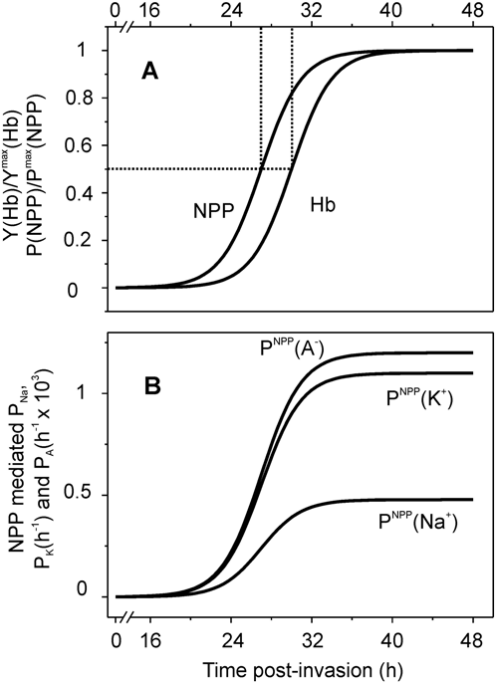
NPP development and Hb consumption as a function of time post-invasion. (A) Normalized time-course of NPP development and of Hb consumption as originally encoded in the model [9],[10] represented as fraction of maximal NPP permeability (P_NPP_/P^max^_NPP_) and maximal digested Hb (Y_Hb_/Y^max^_Hb_), respectively (see equations S21 and 22 in Text S1). The half-time and slope values for the NPP and Hb curves shown here were (in h) 27-2 and 30-2, respectively. (B) cation and anion permeabilities through NPPs as a function of time. Note the difference of three orders of magnitude between cation and anion permeabilities.

Figure 1A illustrates the time courses of NPP development and of Hb consumption, as encoded in the original version of the model, based on the experimental results from Staines et al. [8] and from Krugliak et al. [12], respectively. Figure 1B shows the time-dependent increase in Na^+^, K^+^ and anion permeabilities through the NPP pathway [7]. Note the difference in ordinate scales between anion and cation permeabilities, corresponding to the well-documented anion selectivity of the NPP pathway [7],[19]. We analyse first the rationale behind the two curves in Figure 1A and then the significance of the time shifts between the onset of the NPP-mediated permeabilization and of Hb consumption.

### NPP Development

The stage-dependent changes in NPP-mediated permeabilities were measured in samples from synchronized *Pf* cultures [8]. They were encoded in the model as represented in Figure 1A and 1B. The curves may be interpreted in either of two ways: as a gradual simultaneous increase in NPP-mediated permeability in all the parasitized cells (graded response), or as the net population variation in onset time of sudden permeability changes in individual cells (all-or-none response). Can the available experimental evidence help discriminate between graded or all-or-none alternatives? Isotonic solutions of NPP-permeant solutes such as sorbitol have been extensively used to selectively hemolyse IRBC with developed NPPs [20]. Analysis of the lysis kinetics of IRBCs renders results compatible with both types of responses [21]. Patch-clamp studies have not yet documented intermediate conductance stages in NPP activated IRBCs [22]. Therefore, the all or none response remains a distinct possibility, deserving investigation here by analysing the predicted effects of a sudden increase in NPP-mediated permeability.

### Hemoglobin Consumption and Significance of the Time-Difference between NPP Development and Hemoglobin Consumption

The stage-dependent changes in Hb consumption were defined within wide error margins [12],[23]. Hb consumption of up to 80% of the host cell Hb is known to proceed gradually (see, e.g., [23], and there is no “all or none” alternative to gradual Hb consumption. The most important and well supported feature of the two curves in Figure 1A is that NPP development precedes Hb consumption. Sorbitol, alanine and other solutes whose permeability through uninfected RBC membranes is negligible have been extensively used to probe for NPP permeabilization [7]. In isotonic solutions of sorbitol or alanine, IRBCs with developed NPPs rapidly lyse; only those with young ring-stage parasites remain intact. The importance of delayed Hb digestion relative to NPP development is that by the time the large excess of amino acids produced by globin proteolysis reaches the host cell membrane, the permeability path available for their rapid downgradient exit is available, thus preventing osmotic stress from accumulated amino acids within the host. However, the experimental errors in the observed half-times of NPP development and Hb ingestion curves are relatively large and the possible effects of interval variations between the curves in Figure 1 on IRBC homeostasis deserve exploration.

### IRBC Homeostasis

**Figure 2:**
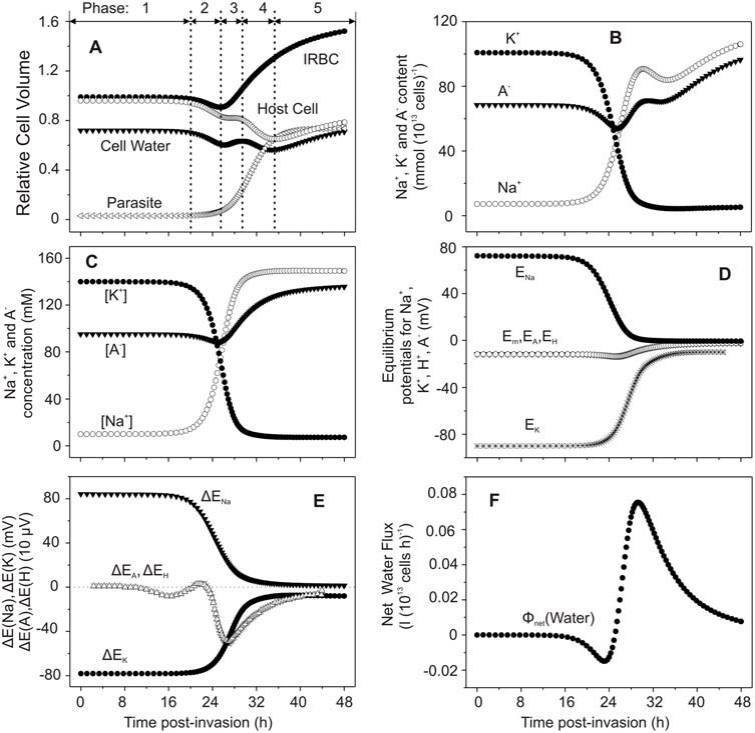
Predicted changes in selected model variables as a function of time post-invasion. The parameter values chosen for this simulation were: coupling factor, 1.0; maximal fraction of Hb consumed, 0.7; half times and slope values for NPP development and Hb consumption curves as in Figure 1 (in h, 27-2 and 30-2). (A) volumes of parasite, host cell water, host RBC and IRBC, relative to IRBC volume at t = 0. (B) Na^+^, K^+^ and diffusible anion content of IRBCs, in mmol (10^13^ cells)^−1^ (10^13^ cells is taken to represent the approximate volume of a litre of normal, uninfected packed red blood cells with a mean cell volume of 100 fl). (C) Na^+^, K^+^ and diffusible anion concentration in host cell water, in mM. (D) Membrane potential (E_m_) and equilibrium potentials (Nernst potentials) of Na^+^ (E_Na_), K^+^ (E_K_), diffusible anion (E_A_) and protons (E_H_) across the host cell membrane. (E) Differences between E_X_ (X = Na^+^ and K^+^ in mV, A^−^ and H^+^ in mV 10^−2^) and E_m_ (ΔE_X_ = E_X_−E_m_), representing the changing driving forces for passive ion fluxes across the host cell membrane throughout the asexual reproduction cycle, in mV. (F) Net water flux across the host cell membrane, in l(10^13^ cells)^−1^(h)^−1^.

**Figure 3:**
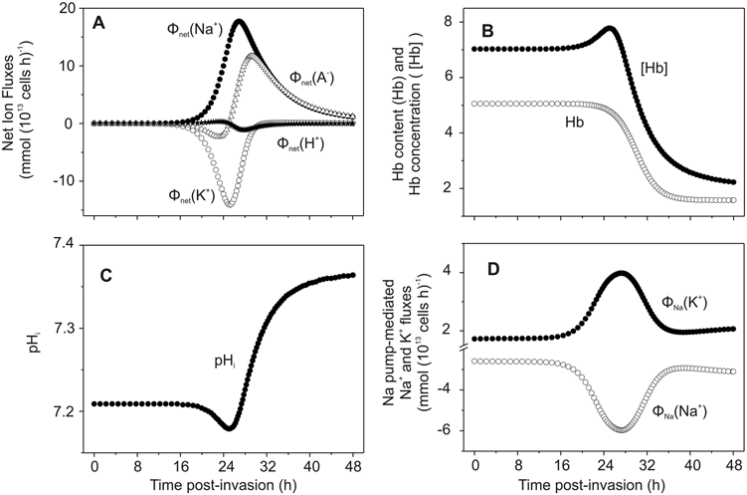
Predicted changes in additional model variables as a function of time post-invasion. (A) Net fluxes of Na^+^, K^+^, A^−^ and H^+^ across the host cell membrane, in mmol(10^13^ cells)^−1^(h)^−1^. (B) Changes in host cell Hb content, in mmol(10^13^ cells)^−1^, and in Hb concentration within host cell water phase, in mM. (C) pH of host cytosol. (D) Na pump-mediated fluxes of Na^+^ and K^+^, in mmol(10^13^ cells)^−1^(h)^−1^.

Figures 2 and 3 show the results of a typical simulation with parameter values chosen for convenient illustration of the five homeostatic periods defined by inflexions in the curves reporting net fluid movements in the host RBC (Phases 1 to 5 in Figure 2A). The model predictions here allow a detailed analysis of the homeostatic processes at work during the different stages of parasite development.

During the stage of *initial quiescence* (Phase 1), from invasion to about 20 h post-invasion, all IRBC variables remain essentially unchanged from their initial levels. Phase 2, *K^+^-driven net fluid loss*, is triggered by NPP activation. The immediate effect of the increase in Na^+^, K^+^ and anion (A^−^) permeabilities (Figure 1B) is to induce the dissipation of the steep initial Na^+^ and K^+^ gradients (Figure 2B and 2C), unrestricted by co-anion movements [24],[25].

Initially the opposite driving forces for Na^+^ and K^+^ gradient dissipation have similar magnitude, as represented in Figure 2D and 2E by the respective electrochemical driving gradients ΔE_Na_ and ΔE_K_. The P_K_/P_Na_ permeability ratio for cation selectivities is however set at 2.3 [8] and thus determines that the loss of KCl transiently exceeds NaCl gain. This causes a transient net fall in RBC cytosolic anion content and concentration (Figure 2B and 2C, respectively) and net loss of water (Figure 2A, cell water and Figure 2F). Figure 3A shows that the K^+^ efflux, which initially exceeds Na^+^ influx, rapidly returns to near-zero baseline levels as the K^+^ gradient is dissipated. The transient dehydration of the IRBC during this second period generates secondary transient changes in other homeostatic variables: increase in Hb concentration (Figure 3B), reduced anion content (Figure 2B, A^−^) and anion concentration (Figure 2C, [A^−^]), and cell acidification (Figure 3C). The transient acidification results from the brief increase in [Cl^−^]_o_/[Cl^−^]_i_ ratio due to net KCl loss; the combined operation of the CO_2_ shunt and anion exchanger rapidly readjusts the proton ratio to restore the equilibrium condition [H^+^]_i_/[H^+^]_o_ = [Cl^−^]_o_/[Cl^−^]_i_, with consequent cell acidification [26]–[28].

In Phase 3, *Na^+^-driven fluid gain*, the direction of net fluid movement is reversed following the reversal of the gradients driving net salt flows. This reversal also affects the direction of change in all associated variables (Figures 2 and 3). Figure 3A shows that the net fluxes of Na^+^ and anions into the cell persist long after the net K^+^ flux has returned to baseline levels, and Figure 2D and 2E shows the time-dependent changes in driving gradients which determine the direction of net ion and fluid fluxes at all times according to the model (see Text S1).

In Phase 4, *fluid loss*, the rate of Hb consumption is maximal. This rate determines the volume of cytosol that the parasite needs to ingest in order to incorporate the amount of Hb prescribed by the Hb consumption function (Figure 1). When this volume exceeds the concomitant Na^+^-driven fluid gain, host cell water contents and host cell volume are transiently reduced (Figure 2A). Phase 4 is characterized by the steepest rates of Hb fall (Figure 3B, Hb) and parasite growth (Figure 2A, open triangles), and by a decline in cell water (Figure 2A, solid triangles). Transient reductions in Na^+^ and anion contents (Figure 2B, Na^+^,A^−^) result from the transfer of RBC cytosol to the parasite as part of the Hb ingestion process. Additional reduction in host cell volume results from the removal of the space occupied by Hb molecules. Hb has a specific volume of about 0.74 ml/g [29] and contributes with about 25% to the total volume of a normal RBC. Therefore, a loss of 70–80% of Hb from a cell containing in average 34 pg of Hb is equivalent to a volume loss of between 15–20 fl by the end of the asexual cycle.

Phase 5, *sustained swelling*, is characterized by continuous NaCl and water gains by the host cell (Figures 2A (cell water), 2B (Na^+^ and A^−^), and 2F) driven by the inward Na^+^ gradient. The rate of fluid gain is reduced relative to that in phase 3 (Figure 2F) because of the marked reduction in driving force for net NaCl gain (Figure 2D and 2E) and in colloid-osmotic pressure due to the fall in Hb concentration (Figure 3B). Parasite and IRBC volumes also increase at slower rates (Figure 2A (Parasite, IRBC)) following the reduced Hb consumption and fluid gains relative to Phase 4. As the anion concentration increases (Figure 2C), the membrane potential becomes progressively more depolarized and the equilibrium potentials of all ions approach the membrane potential E_m_ (Figure 2D and 2E), with consequent cell alkalinisation (Figure 3C). The Na pump, initially stimulated by the increased intracellular Na^+^ concentration, shows late inhibition (Figure 3D). This inhibition results from a predicted reduction in Mg^2+^ concentration in the host cell cytosol following global cytosolic transfers to the parasite during Hb ingestion. Late swelling further reduces the Mg^2+^ concentration. The Mg^2+^/ATP ratio is an important regulator of Na pump activity and departure from its normal value near unity is inhibitory to the pump [30],[31]. Atamna and Ginsburg [32] measured the Mg^2+^ content of host and parasite compartments in IRBCs with mature trophozoite stage parasites and found that the Mg^2+^ content of the host cell compartment was over 60% lower than that of uninfected RBCs. They suggested that such a reduction may partially inhibit active transport by the sodium and calcium pumps. It remains to be elucidated whether the actual mechanism of Mg^2+^ deprivation in the host cell is the one implied by the model.

**Figure 4:**
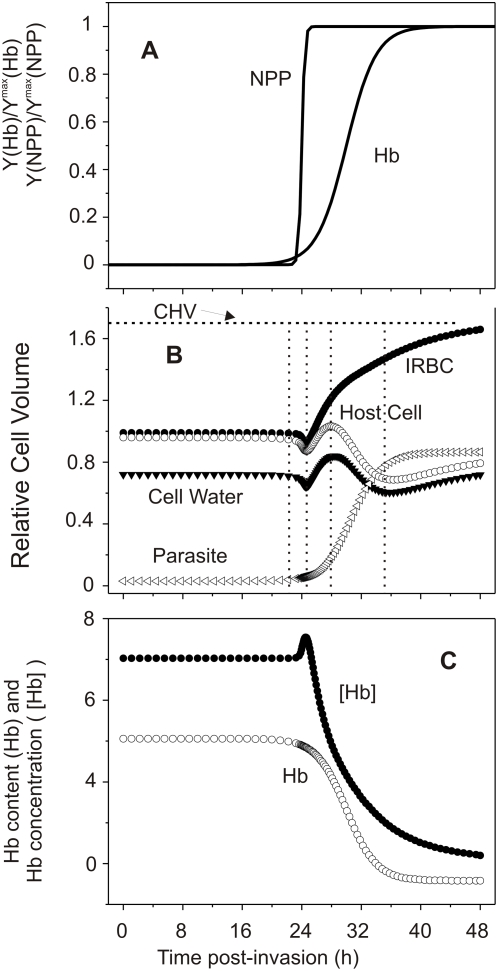
Time dependent effects of all-or-none NPP activation on selected model variables. (A) Normalized curves representing the time-course of NPP development and of Hb consumption applied in this simulation. The half-time and slope values for the NPP and Hb curves shown here were (in h) 24-0.2 and 32-2, respectively. (B and C) As for Figures 2A and 3B respectively. Following Ponder [35], the critical hemolytic volume shown was set at 1.7 times the relative cell volume.

Figure 4 illustrates a condition in which NPPs are switched on almost instantly to analyse the homeostatic effects of all-or-none NPP activation (Figure 4A). The rest of parameter values were the same as for Figure 2. It can be seen that the main effect of all-or-none NPP activation is to compress the time-course of the events described for phase 2 (Figure 2), with relatively minor long-term quantitative changes in volumes (Figure 4B) and in Hb concentration (Figure 4C). The relative duration and magnitude of the effects described for each period in the examples chosen for Figures 2 and 4 will vary with the choice of parameter values. These effects are analysed below. The important point to note is that the underlying homeostatic processes described for each period remain essentially the same.

### Design of a Modelling Strategy for Parasite Volume Growth; Introduction of a Coupling Factor Linking Parasite Volume Growth to Hemoglobin Consumption

The time course of volume growth of *P. falciparum* parasites throughout their asexual reproduction cycle in human RBCs has not been characterized. Parasite volume increases throughout the cycle but it is unknown whether this growth is uniform or variable. The minimal final parasite volume in a cell with a single parasite has to equal the sum of the volumes of all the merozoites produced plus the volume occupied by the residual body. Without relevant information available, it was difficult to design a rational strategy to model parasite volume growth. Because the time-course of parasite volume growth could be roughly associated with that of Hb ingestion, linking these two variables was considered an acceptable modelling strategy. In the initial formulation of the model [9], parasite volume at each instant of time was defined by the cumulative volume of ingested host cell cytosol up to that time. This volume, in turn, was determined by all the complex homeostatic factors that influenced the volume of host cytosol in which the prescribed amount of Hb to be digested at each time was contained. For maximal Hb consumption around 70–80%, this strategy predicted terminal parasite volumes of about 70 to 90 fl (Figure 2A, Parasite), values near the mean volume of uninfected RBCs. However, previous results suggested that this approach overestimated parasite volume.

**Figure 5:**
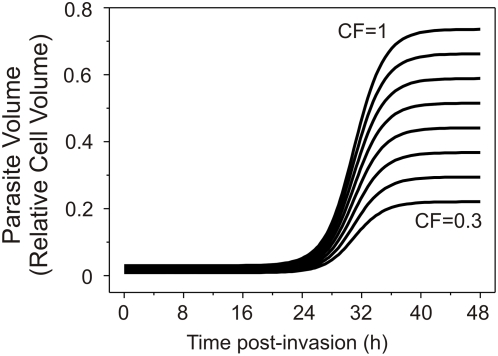
Effect of coupling factor on the magnitude of parasite volume-growth. The time-course of parasite volume growth was linked to the ingestion of host cytosol. The volume of cytosol incorporated into the parasite at each instant of time is determined by the need to provide the right amount of Hb, as prescribed by the Hb consumption function (equation S22 in Text S1). The coupling factor defines the fraction by which the cumulative cytostomal ingestions convert to parasite volumes (equation S28 in Text S1). With a coupling factor of 1, parasite volume growth reflects the cumulative sum of all cytostomal ingestions. It can be seen that with a maximal Hb consumption set at 70% in the present simulation, parasite volume nears 80% of the original host cell volume. In the curves shown here the coupling factor was varied from 0.3 to 1.0 in steps of 0.1. All other parameters are as in Figures 2 and 3.

Saliba et al. [33] measured the water content of parasites at the mature trophozoite stage to be less than 30 fl. Recent results by Elliott et al. [23] suggest that single parasite volumes seldom exceed 50 fl at any developmental stage. Therefore, to explore the effect of more realistic estimates of parasite volumes a coupling factor was introduced. It defines the global volume-growth of the parasite in each iteration of the numerical computation as a fraction of the volume of cytosol incorporated during that iteration (see Text S1). For coupling factor values of less than 0.7, this approach implicitly corrects for parasite volume losses due to Hb breakdown, because, although hemozoin is retained, the volume occupied by the globin molecules largely vanishes in the process of exporting the resulting amino acids to the external medium. As explained above, this volume may account for up to 20 fl. The results of simulations using the same set of parameter values applied in the example of Figure 2, varying only the value of the coupling factor, are shown in Figure 5 which reports predicted parasite volumes as a function of time post-invasion. From these results, only coupling factor values in the range 0.3 to 0.7 appear to cover the observed range of terminal parasite volumes for single infections. This range then will be tested in the global simulations attempted below, in comparison with the original value of 1 [9],[10].

### Model Predictions over the Range of Variation in Parameter Values

**Figure 6:**
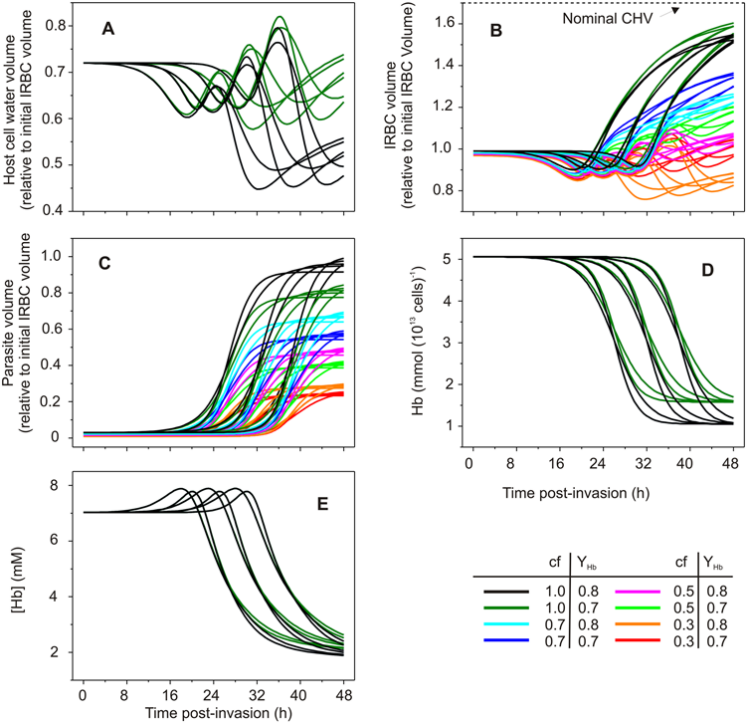
Effect of parameter variation on predicted changes as a function of time post-invasion. The parameters varied and the range of values reported in the 48 curves of this figure are as follows: coupling factor (cf), 0.3, 0.5, 0.7 and 1; paired half-times of NPP development and Hb consumption (in h), 22–26, 27–32, and 32–38; slope-parameter of NPP and Hb curves (in h), 2 and 3; maximal Hb consumption (fraction of total initial Hb), 0.7 and 0.8. (A): host cell water volume, relative to initial IRBC volume; (B): IRBC volume, relative to initial volume. The broken line represents the nominal CHV based on a mean value of 1.7 reported for normal uninfected RBCs [35]; (C): relative parasite volume; (D): host cell Hb content, in mmol(10^13^ cells)^−1^; (E): Hb concentration in host cell water, mM. Overlapping curves in Panels A, D and E conceal all the 48 curves within the 12 that can be discerned.

Figure 6 shows the model predictions for five selected variables, plotted as a function of time post-invasion. The chosen range of variation for each parameter was based on experimental results when available or on outcomes of simulations consistent with observation. For instance, although single parasite volumes may remain within the 30 to 50 fl range, IRBCs are often seen with two viable parasites reaching segmentor stages (L. Bannister, personal communication), or with additional volume occupied by developmentally arrested parasites [34]. From the perspective relevant to the homeostasis of the host cell, it is the combined parasite volume that counts, hence the choice of coupling ratios spanning values from 0.3 to 1. The range of half-time values for NPP development and Hb ingestion is shown within ±1 standard deviation of the experimentally-reported means [8],[12]. Figure 6 shows only simulations with parameter values in which IRBC volumes remain below the spherical volume cells can attain within a maximally-stretched membrane, at which point they would lyse. This maximal volume is usually described as the critical hemolytic volume. Following Ponder [35], the nominal CHV was set at a mean value of 1.7 times the original volume of the modelled cell.

The immediate conclusion from gross comparisons between Figure 6A–C and Figure 6D and 6E is that whereas host cell water (Figure 6A), IRBC volume (relative to uninfected RBC volume, Figure 6B), and parasite volumes (Figure 6C) can vary over a very wide range and with large oscillations within the five homeostatic periods described for Figure 2 (Figure 6A and 6B), the predicted decline pattern in host cell Hb concentration remains remarkably uniform (Figure 6E). Therefore, the single novel and invariant prediction of the colloid-osmotic hypothesis is that the Hb concentration within the host cell has to become progressively reduced, regardless of parasite and IRBC volumes (Figure 6E).

## Discussion

The analysis of the homeostasis of *Pf* infected RBCs (Figures 2, 3, 4, and 6) provides a number of novel insights:

- it identifies five distinct periods associated with dynamic changes in ionic driving gradients, in hemoglobin and ion contents, in ionic fluxes through NPPs and Na pump, and in water fluxes (Figures 2 and 3);
- it predicts specific stage-dependent variations in host cell pH (Figure 3C) and membrane potential (Figure 2D);
- it reveales a wide domain of homeostatic stability compatible with large variations in parasite and IRBC volumes throughout the asexual reproduction cycle of the parasite (Figure 6), and
- it demonstrates the homeostatic compatibility of an all-or-none mode of activation of NPPs (Figure 4). The graded and all-or-none alternatives for NPP activation in single IRBCs are relevant to ongoing controversies on whether NPPs represent upregulated endogenous pathways of the RBC membrane or whether they are parasite-induced insertions [22],[36],[37]. Upregulation, as recently suggested [22], could explain better an all-or-none modality by a developmental-stage controlled release of a NPP-activation factor within each IRBC. However, the all-or-none alternative remains untested experimentally. One possible approach would be to measure NPP-mediated conductance in stage-segregated IRBCs using the cell-attached patch clamp configuration. Graded NPP development predicts a clear trend of increasing conductance with parasite developmental stage whereas all-or-none predicts similar large NPP conductances at all developmental stages whenever detected. We now discuss some of the issues arising from this analysis.

**Figure 7:**
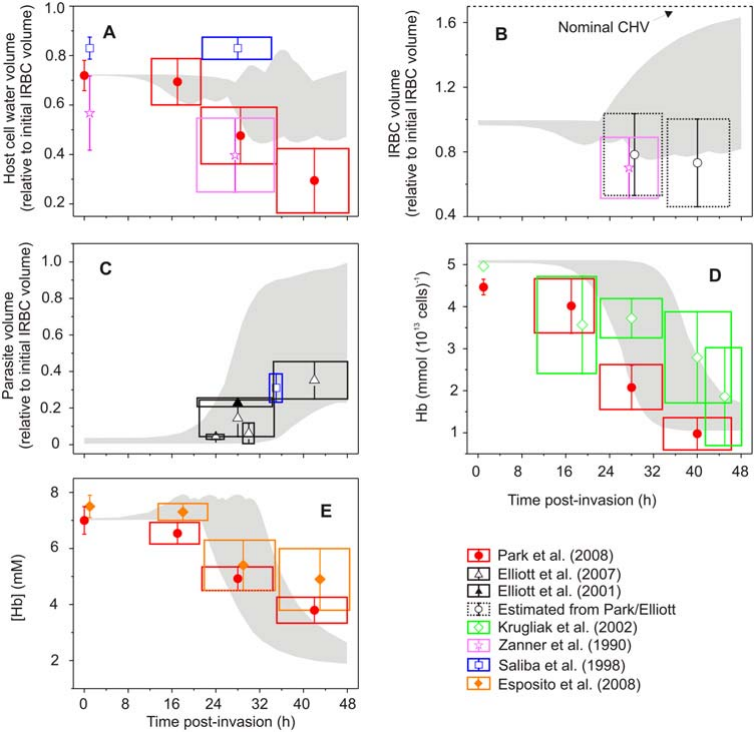
Comparison between predicted and measured variable values. Measured values were obtained from the references listed. The ranges of predicted variable values shown in Figure 6 are redrawn as grey silhouettes; the experimental data is plotted as boxes. On the ordinate the box indicates the reported statistical errors; on the time axis the box width is arbitrarily set to 26–34 h for trophozoites, 36–46 h for schizonts, and 42–48 h for segmentors using the period assignations in the original papers. Data for uninfected cells is shown at time = 0, where available. Symbols indicate the source of the data, as follows: closed circles – Park et al. [40]; open and closed triangles – Elliott et al. [23],[38]; open diamonds – Krugliak et al. [12]; open stars – Zanner et al. [39]; open squares – Saliba et al. [33]; closed diamonds – Esposito et al. [41]. (A) Host cell water volume, relative to initial IRBC volume; (B) IRBC volume, relative to initial volume; (C) relative parasite volume; (D) host cell Hb content, in mmol(10^13^ cells)^−1^; (E) Hb concentration in host cell water, mM. Data in (B) and (E) were modified from the original references to fit the units used in the model (E) or to provide plausible estimates in the absence of direct measurements (B). IRBC volumes in (B) (dotted lines) were estimated from data in Park et al. [40] and Elliot et al. [23] by adding the cytosol volume from Park et al. [40] to the parasite volume from Elliot et al. [23]. [Hb] data in Panel E, from Park et al. [40], was obtained by dividing the Hb content of the cytosol by the water content of the cytosol after subtracting the volume occupied by the Hb molecules. The data was then normalized to the initial value of [Hb] used for the model simulations.

We consider next how experimental results in the literature compare with our model predictions. In Figure 7, experimental measurements of the five selected variables reported in Figure 6 are shown as rectangles over the grey silhouettes of the variable ranges in Figure 6. Despite the large variability in the experimental results, it is clear that they fall within the low range of values for host cell water (Figure 7A), IRBC volume (Figure 7B), and parasite volume (Figure 7C) for single infections [33],[38],[39]. The hemoglobin content measurements show a declining pattern covering the full range of the values encoded in the model (Figure 7D). Most significantly, the recent measurements of the stage-dependent changes in host Hb concentration by Park et al. [40] and Esposito et al. [41], obtained with two independent techniques confirm the declining pattern predicted by the model (Figure 7E), thus lending support to its most relevant prediction.

Do the large variations in parasite and host cell volumes explored with the model and also apparent in the experimental measurements (Figure 7) reflect true IRBC polymorphisms or merely error margins? IRBC polymorphisms are evident in relation to a number of characteristics which can be easily observed and recorded in live cultures: single or multiple invasion, developmental and viability differences among parasites in multiple invasions, IRBC shapes and volumes, parasite sizes and shapes, hemozoin particle content, aggregation state of hemozoin crystals, number of merozoites contained and released, etc. Many of these variations are observed in highly synchronized populations and cannot be attributed simply to differences in developmental stage of the parasite, or to viability state of the IRBCs. It is therefore plausible that the domain of stable homeostatic solutions predicted by the model (Figure 6) does indeed reflect, at least in part, true homeostatic polymorphisms of IRBCs. If so, parasite, host and IRBC volumes may vary within wide margins from cell to cell without necessarily compromising the osmotic stability of the IRBC. This, however, questions the fundamental tenet of the colloid-osmotic hypothesis, that excess Hb consumption is necessary to prevent premature IRBC lysis.

The problem can be clearly illustrated with an example. Let us consider an IRBC whose volume remains near a relative cell volume of 1 throughout the asexual reproduction cycle, as in many of the curves shown in Figure 6B, and as documented experimentally [23],[33],[40]. If the critical hemolytic volume remains set at 1.7 times the initial RBC volume, as originally assumed based on data from uninfected RBCs [35], model simulations indicate that ∼20% Hb consumption would be enough to prevent the premature lysis of a cell with relative volume around 1. So, for such a cell, excess Hb consumption would appear irrelevant for lysis prevention. But the available evidence overwhelmingly supports the view that Hb is consumed in large excess in all viable IRBCs. It follows that reduced colloid-osmosis may not be the main reason for excess Hb consumption, at least not in all instances. However, as discussed next, the conundrum here rests with the rigid attribution of a CHV of 1.7 in the model simulations, not with the basic understanding of IRBC homeostasis provided by the model.

Previous results from osmotic fragility studies in IRBCs showed that the osmotic fragility of RBCs infected with mature trophozoite- and schizont-stage parasites is substantially increased relative to IRBCs with ring-stage parasites or to uninfected cohorts [9],[10]. If the actual volume of at least some IRBCs harbouring mature parasites remains low, then for the osmotic fragility to be increased their CHV has to be somehow reduced.

How can this occur? The CHV of each RBC depends critically on membrane area [35],[42],[43]. If membrane area is reduced by infection, the CHV will also be reduced. The relation between volume (V) and area (A) in a sphere is given by V = A^3/2^/(6π^1/2^). Therefore, since the ratio of maximal volumes (V_1_, V_2_) of two cells with different surface areas (A_1_, A_2_) is V_1_/V_2_ = (A_1_/ A_2_)^1.5^, a fractional decrease in area will propagate to a fractional decrease in volume to the power of 1.5, stressing the magnified effect of effective membrane area reductions on CHV. Early results in the literature report opposing claims in relation to membrane area changes in infected IRBCs: population estimates report substantial reductions [39] whereas single cell measurements suggested no change [44]. Recent movies by Glushakova et al. [45] show infected cells about to rupture whose near spherical diameter is less than 80% that of surrounding uninfected discocytes indicating reduced membrane area. Considering, in addition, that the membrane geometry and fluid properties of Pf-infected RBCs become progressively altered by knobs and increased rigidity [46]–[51], the increased osmotic fragility may be compounded by an increased lytic vulnerability to volume expansion, resulting from reductions in membrane area, in the capacity to effect a normal expansion of the full membrane area before lytic rupture, or both.

The increase in the osmotic fragility of IRBCs reflects a progressive hemolytic vulnerability of *Pf*-infected RBCs to volume expansion by fluid gains. This shows that IRBCs become progressively closer to their effective CHV as the parasite matures, regardless of their actual volume levels. Although the contributions of membrane area loss and other factors to this hemolytic vulnerability remain to be elucidated, excess Hb consumption retains its credential as a general protection mechanism for IRBCs of all volumes, by preventing excessive rates of fluid gain. This mechanism depends critically on the prediction illustrated in Figures 3B and 6E that the Hb concentration must become progressively reduced in all IRBCs, regardless of the specific volume evolution of each IRBC.

In conclusion: the original formulation of the colloid-osmotic hypothesis, using a coupling coefficient of one, predicted that IRBCs would swell close to a CHV level taken as the mean value for uninfected RBCs, premature rupture being prevented by the reduced Hb concentration. Simulations with coupling coefficient values below 0.7 deliver more realistic IRBC volume estimates (Figures 5B and 7B), but experimental results indicate that the progressive proximity of IRBCs to a reduced CHV is retained. Thus, whatever the reason for CHV proximity, reduction in Hb concentration remains essential for preventing rapid fluid gains leading to premature IRBC lysis.

## Model

### The Software

The mathematical-computational model of the homeostasis of *Plasmodium falciparum*-infected red blood cells (IRCM) was derived as an extension of the original Lew-Bookchin red cell model (RCM) [26]. Both models are available as free-standing executable files from http://www.pdn.cam.ac.uk/staff/lew/index.html. The IRCM first computes a “Reference” steady-state (RS) meant to represent the initial condition of a human red blood cell just invaded by a *falciparum* merozoite generating a ring-stage internalized parasite occupying 4% of the red cell volume. In the formulation of the RS the programme offers a large variety of options for the user to change constitutive properties of the IRBC such as the value of all the parameters tested in the simulations reported in this paper. For the simulations reported here the medium was assumed to be an infinite reservoir (vanishingly low hematocrit condition). With the RS defined, the programme is set to follow the dynamic evolution of the IRBC system (Dynamic state, DS) for 48 hours with a data output frequency chosen by the user. To enable realistic comparisons with experimental results, experimental conditions can be simulated to explore the modified dynamic behaviour of the system.

### The IRBC Model

#### Computation of the reference state

The constitutive properties of IRBCs defined in the RS comprise (see supporting information for equations):

1. The detailed initial composition of intra and extracellular fluids complying with electroneutrality and osmotic equilibrium (eqs S1 and S2)
2. The identity, concentration, and protonized state of medium H^+^ buffers (HEPES-like in the current simulations (eq S3)
3. The identity, concentration and mean charge of the impermeant cell solutes (eqs S4 and S5)
4. The cytoplasmic H^+^ buffering behaviour of Hb (eq S5 ) [52]
5. The power function describing the non-ideal osmotic behaviour of Hb (eq S6) [53],[54]
6. The initial fraction of IRBC volume occupied by Hb molecules (25%) [29],[55]
7. The magnitude and kinetics of active Na^+^ and K^+^ transport through the sodium pump (eqs S7–S10) [55],[56]
8. The magnitude and kinetics of all unidi^r^ection^a^l and net passive fluxes of Na^+^, K^+^, A^−^ and H^+^ through each of the endogenous passive transporters expressed in the cell (eqs S11–S18) [26]
9. The electrical potential potential difference across the RBC membrane (eqs S19,S20)
10. The stage-dependent changes in NPP-mediated permeabilities (Figure 1, eq S21) [7]–[9]
11. The maximal magnitude and kinetics of Na^+^, K^+^ and A^−^ fluxes through NPPs (eq S13) [7]
12. The Hb consumption function (Figure 1, eq S22) [9]
13. Cell volume normalized to 1 at t = 0 [55]
14. Area-volume ratio of RBC encoded in critical hemolytic volume set at 1.7 times the initial cell volume (volume of sphere contained within original RBC membrane area) [35]

All functions were defined with a default set of parameter values, open to change by the modeller. The default values are displayed at the prompts in the running of the model. The initial values of most system parameters and variables were listed following a hierarchical order of reliability for the information available so as to enable derivation of lesser known from better known values in the equations of the Reference State [26],[55]. The kinetic equations were defined with minimal sets of parameters to avoid unnecessary complexity.

#### Computation of the dynamic state

Three main processes determine the overall dynamic behaviour of IRBCs: NPP-mediated traffic, Hb consumption and parasite volume growth. The potential osmotic stress generated by the amino acids produced during Hb digestion was neglected in the model because the amino acid composition of the effluent from infected RBCs was indistinguishable from that of globin, indicating that NPP permeability was high enough not to limit the rate of exit of the lesser permeable amino acids [7],[11],[12].

Parasite volume growth was linked to Hb ingestion as follows. For any infinitesimal time interval, represented by the integration interval D*t* in the numerical computations, the parasite ingests a volume of red cell cytoplasm, D*V_w_* (eqs S26, S27) containing the amount of Hb prescribed by the Hb consumption function for that time interval (eq S22). This reduces the host cell volume by D*V_w_* and increases parasite volume by *cf*·D*V_w_* in each D*t*. *cf* represents a coupling coefficient which converts D*V_w_* to a quantum of parasite volume-growth within D*t*, proportional to D*V_w_*, to enable the modeller to explore the effect of broad parasite volume-growth variations, and also the volume-effects of single and multiple invasions. Global volume changes within the host compartment are determined by parasite ingestion of host cytosol and by fluid transfers across the host cell plasma membrane resulting from changes in membrane permeability (NPP development), ionic gradients and colloid osmosis. Ingestion, parasite volume growth and homeostasis are therefore closely interdependent processes because ingestion affects host Hb content on which colloid osmosis largely depends and homeostasis affects the size of each D*V_w_* on which parasite volume growth depends. At each time, *t*, parasite volume, *V_P_*, and host cell volume, *V_H_*, result from the cumulative changes generated by host cytosol transfers to parasite and by all the ion and water fluxes through endogenous and parasite-generated pathways up to *t*. Thus, *V_P_* = *V_P_^o^*+*cfΣ* D*V_w_* and *V_H_* = *V_H_^o^*+*Σ*D*V'*−*Σ* D*V_w_*, respectively, where *V_P_^o^* represents the initial ring-stage parasite volume, *V_H_^o^* represents the initial host cell volume, normalized to 1, and D*V'* represents the homeostasis-induced infinitesimal change in host cell volume within each D*t*. In the running of the model, *V_H_* at each *t* is computed from equation S30, as detailed in Text S1. IRBC volume at each *t*, *V_IRBC_*, is thus defined by *V_IRBC_* = *V_H_*+*V_P_*. Solute exchanges between parasite and host, as well as possible homeostatic effects resulting from host cell membrane alterations were not considered in the model because their relevance remains uncertain and precise information, with the detail required for useful modelling is lacking.

The following sequence was applied in the computation of the transient behaviour of the IRCM for each D*t* interval.

1. RS defined
2. If simulating experiments, enter changes in medium composition and system parameter values
3. Compute membrane potential from equation S20, implicit in *E_m_*
4. With *E_m_* defined, compute net fluxes through all transporters, *Φ_i_* (eqs S7–S17)
5. With *Φ_i_* defined, compute the change in cell amount of each solute, D*Q_i_* (eqs S23 and S24) and the new amount of solute i, *Q_i_^t^* (eq S25)
6. Compute the volume of RBC cytosol, D*V_W_* (eq S26), to be ingested during D*t* to provide the amount of Hb to the parasite specified by equations S22 and S27 for each *t*
7. Compute parasite volume, *V_P_* (eq S28)
8. Compute the new concentrations of each cell solute C_i_ (eq S29)
9. Compute the water flux, *Φ_W_* (eq S18)
10. Compute the new volume of host cell water, *V_w_* (eq S27)
11. Compute the new host cell volume, *V_H_* (eq S30, S31)
12. Compute the new value of the iteration interval, D*t* (eq S32)
13. Convert the new *C_H_* to *pH^c^* (eq S33), compute the new charge on Hb, *n_Hb_* (eq S4), and the new anion and proton concentration ratios (eqs S34 and S35, respectively)
14. Compute the new *f_Hb_* from the new *C_Hb_* (eq S6)
15. Test for *t* assigned to output results (if no, continue; if yes, output results)
16. Test for IRBC volume exceeding set value of critical hemolytic volume (if no, continue; if yes, terminate programme and output final results)
17. Test for *t* = 48 h (if no, continue; if yes, terminate programme and output final results)
18. Test for further input of experimental conditions (if no, continue; if yes, stop, input further changes, continue)
19. Go to step 3

## Supporting Information

Text S1 Model equations and glossary of symbols
(0.14 MB PDF)

## References

[1] Ginsburg H, Stein WD (1987). New permeability pathways induced by the malarial parasite in the membrane of its host erythrocyte: potential routes for targeting of drugs into infected cells.. Biosci Rep.

[2] Ginsburg H, Benga GH, Trager JM (1988). How and why does the malarial parasite permeabilize its host cell membrane?. Biomembranes: Basic & Medical Research..

[3] Kanaani J, Ginsburg H (1988). Compartment analysis of ATP in malaria-infected erythrocytes.. Biochem Int.

[4] Kanaani J, Ginsburg H (1989). Metabolic interconnection between the human malarial parasite Plasmodium falciparum and its host erythrocyte. Regulation of ATP levels by means of an adenylate translocator and adenylate kinase.. J Biol Chem.

[5] Kanaani J, Ginsburg H (1991). Transport of lactate in Plasmodium falciparum-infected human erythrocytes.. J Cell Physiol.

[6] Kirk K, Staines HM, Martin RE, Saliba KJ (1999). Transport properties of the host cell membrane.. Novartis Found Symp.

[7] Kirk K (2001). Membrane transport in the malaria-infected erythrocyte.. Physiol Rev.

[8] Staines HM, Ellory JC, Kirk K (2001). Perturbation of the pump-leak balance for Na^+^ and K^+^ in malaria- infected erythrocytes.. Am J Physiol Cell Physiol.

[9] Lew VL, Tiffert T, Ginsburg H (2003). Excess hemoglobin digestion and the osmotic stability of Plasmodium falciparum-infected red blood cells.. Blood.

[10] Lew VL, Macdonald L, Ginsburg H, Krugliak M, Tiffert T (2004). Excess haemoglobin digestion by malaria parasites: a strategy to prevent premature host cell lysis.. Blood Cells Mol Dis.

[11] Zarchin S, Krugliak M, Ginsburg H (1986). Digestion of the host erythrocyte by malaria parasites is the primary target for quinoline-containing antimalarials.. Biochem Pharmacol.

[12] Krugliak M, Zhang J, Ginsburg H (2002). Intraerythrocytic Plasmodium falciparum utilizes only a fraction of the amino acids derived from the digestion of host cell cytosol for the biosynthesis of its proteins.. Mol Biochem Parasitol.

[13] Ginsburg H, Kutner S, Krugliak M, Cabantchik ZI (1985). Characterization of permeation pathways appearing in the host membrane of Plasmodium falciparum infected red blood cells.. Mol Biochem Parasitol.

[14] Ginsburg H, Krugliak M, Eidelman O, Cabantchik ZI (1983). New permeability pathways induced in membranes of Plasmodium falciparum infected erythrocytes.. Mol Biochem Parasitol.

[15] Orjih AU, Banyal HS, Chevli R, Fitch CD (1981). Hemin lyses malaria parasites.. Science.

[16] Ridley RG, Dorn A, Vippagunta SR, Vennerstrom JL (1997). Haematin (haem) polymerization and its inhibition by quinoline antimalarials.. Ann Trop Med Parasitol.

[17] Ginsburg H, Ward SA, Bray PG (1999). An integrated model of chloroquine action.. Parasitol Today.

[18] Allen RJ, Kirk K (2004). Cell volume control in the Plasmodium-infected erythrocyte.. Trends Parasitol.

[19] Ginsburg H, Stein WD (2004). The new permeability pathways induced by the malaria parasite in the membrane of the infected erythrocyte: comparison of results using different experimental techniques.. J Membr Biol.

[20] Lambros C, Vanderberg JP (1979). Synchronization of Plasmodium falciparum erythrocytic stages in culture.. J Parasitol.

[21] Krugliak M, Ginsburg H (2006). The evolution of the new permeability pathways in Plasmodium falciparum-infected erythrocytes—a kinetic analysis.. Exp Parasitol.

[22] Bouyer G, Egee S, Thomas SL (2007). Toward a unifying model of malaria-induced channel activity.. Proc Natl Acad Sci U S A.

[23] Elliott DA, McIntosh MT, Hosgood HD, Chen S, Zhang G (2008). Four distinct pathways of hemoglobin uptake in the malaria parasite Plasmodium falciparum.. Proc Natl Acad Sci U S A.

[24] Ginsburg H, Handeli S, Friedman S, Gorodetsky R, Krugliak M (1986). Effects of red blood cell potassium and hypertonicity on the growth of Plasmodium falciparum in culture.. Z Parasitenkd.

[25] Lee P, Ye Z, Van Dyke K, Kirk RG (1988). X-ray microanalysis of Plasmodium falciparum and infected red blood cells: effects of qinghaosu and chloroquine on potassium, sodium, and phosphorus composition.. Am J Trop Med Hyg.

[26] Lew VL, Bookchin RM (1986). Volume, pH and-ion content regulation in human red cells: analysis of transient behavior with an integrated model.. J Membr Biol.

[27] Lew VL, Bookchin RM (2005). Ion transport pathology in the mechanism of sickle cell dehydration.. Physiol Rev.

[28] Freeman CJ, Bookchin RM, Ortiz OE, Lew VL (1987). K-permeabilized human red cells lose an alkaline, hypertonic fluid containing excess K over diffusible anions.. J Membr Biol.

[29] Bureau M, Banerjee R (1976). Structure-volume relationships in hemoglobin. A densitometric and dilatometric study of the oxy leads to deoxy transformation.. Biochimie.

[30] Garrahan PJ, Glynn IM (1967). Factors affecting the relative magnitudes of the sodium:potassium and sodium:sodium exchanges catalysed by the sodium pump.. J Physiol.

[31] Flatman PW, Lew VL (1979). The magnesium-dependence of sodium:potassium and sodium:sodium exchange mediated by the sodium pump in intact human red cells.. J Physiol.

[32] Atamna H, Ginsburg H (1997). The malaria parasite supplies glutathione to its host cell. Investigation of glutathione transport and metabolism in human eryhtrocytes infected with Plasmodium falciparum.. Eur J Biochem.

[33] Saliba KJ, Horner HA, Kirk K (1998). Transport and metabolism of the essential vitamin pantothenic acid in human erythrocytes infected with the malaria parasite Plasmodium falciparum.. J Biol Chem.

[34] Simpson JA, Silamut K, Chotivanich K, Pukrittayakamee S, White NJ (1999). Red cell selectivity in malaria: a study of multiple-infected erythrocytes.. Trans R Soc Trop Med Hyg.

[35] Ponder E (1948). Hemolysis and Related Phenomena.

[36] Desai SA, Bezrukov SM, Zimmerberg J (2000). A voltage-dependent channel involved in nutrient uptake by red blood cells infected with the malaria parasite [see comments].. Nature.

[37] Staines HM, Alkhalil A, Allen RJ, De Jonge HR, Derbyshire E (2007). Electrophysiological studies of malaria parasite-infected erythrocytes: current status.. Int J Parasitol.

[38] Elliott JL, Saliba KJ, Kirk K (2001). Transport of lactate and pyruvate in the intraerythrocytic malaria parasite, Plasmodium falciparum.. Biochem J.

[39] Zanner MA, Galey WR, Scaletti JV, Brahm J, Vander Jagt DL (1990). Water and urea transport in human erythrocytes infected with the malaria parasite Plasmodium falciparum.. Mol Biochem Parasitol.

[40] Park Y, Diez-Silva M, Popescu G, Lykotrafitis G, Choi W (2008). Refractive index maps and membrane dynamics of human red blood cells parasitized by Plasmodium falciparum.. Proc Natl Acad Sci U S A.

[41] Esposito A, Tiffert T, Mauritz JMA, Schlachter S, Bannister L (2008). FRET imaging of Hemoglobin Concentration in Plasmodium falciparum-infected red cells.. PLoS ONE.

[42] Lew VL, Raftos JE, Sorette MP, Bookchin RM, Mohandas N (1995). Generation of normal human red cell volume, hemoglobin content and membrane area distributions, by “birth” or regulation?. Blood.

[43] Svetina S (1982). Relations among variations in human red cell volume, density, membrane area, hemoglobin content and cation content.. J Theor Biol.

[44] Nash GB, O'Brien E, Gordon-Smith EC, Dormandy JA (1989). Abnormalities in the mechanical properties of red blood cells caused by Plasmodium falciparum.. Blood.

[45] Glushakova S, Yin D, Li T, Zimmerberg J (2005). Membrane transformation during malaria parasite release from human red blood cells.. Curr Biol.

[46] Suresh S, Spatz J, Mills JP, Micoulet A, Dao M (2005). Connections between single-cell biomechanics and human disease states: gastrointestinal cancer and malaria.. Acta Biomater.

[47] Paulitschke M, Nash GB, Anstee DJ, Tanner MJ, Gratzer WB (1995). Perturbation of red blood cell membrane rigidity by extracellular ligands.. Blood.

[48] Paulitschke M, Nash GB (1993). Membrane rigidity of red blood cells parasitized by different strains of Plasmodium falciparum.. J Lab Clin Med.

[49] Paulitschke M, Preece A, Nash GB (1994). Effect of neuraminidase on rigidity of the red cell membrane.. Biorheology.

[50] Glenister FK, Coppel RL, Cowman AF, Mohandas N, Cooke BM (2002). Contribution of parasite proteins to altered mechanical properties of malaria-infected red blood cells.. Blood.

[51] Glenister FK, Fernandez KM, Kats LM, Hanssen E, Mohandas N (2009). Functional alteration of red blood cells by a megadalton protein of Plasmodium falciparum.. Blood.

[52] Cass A, Dalmark M (1973). Equilibrium dialysis of ions in nystatin-treated cells.. Nature New Biol.

[53] McConaghey PD, Maizels M (1961). The osmotic coefficients of haemoglobin in red cells under varying conditions.. J Physiol.

[54] Freedman JC, Hoffman JF (1979). Ionic and osmotic equilibria of human red blood cells treated with nystatin.. J Gen Physiol.

[55] Lew VL, Freeman CJ, Ortiz OE, Bookchin RM (1991). A mathematical model on the volume, pH and ion content regulation of reticulocytes. Application to the pathophysiology of sickle cell dehydration.. J Clin Invest.

[56] Garrahan PJ, Garay RP (1974). A kinetic study of the Na pump in red cells: its relevance to the mechanism of active transport.. Ann N Y Acad Sci.

