# Supplementary material for: The Homeostasis of Plasmodium falciparum-Infected Red Blood Cells
Source: PLoS Comput Biol. 2009 Apr 3;5(4):e1000339. doi: 10.1371/journal.pcbi.1000339 (PMC2659444; doi:10.1371/journal.pcbi.1000339)
Supplement: Text S1 — Model equations and glossary of symbols (0.14 MB PDF) [file pcbi.1000339.s001.pdf]

Text S1

Glossary of symbols

The nomenclature follows that applied in earlier models of the homeostasis of erythroid cells [1,2]. No distinctions are made between Cl^- and other highly permeable anions (i.e. HCO_3^-), globally labelled A. Parameters of the Na:Cl and K:Cl cotransports are indexed NaA, KA, respectively, in the understanding that only Cl^- is meant here, whereas for the Cl:H cotransport representing the operation of the Jacobs-Stewart mechanism [2], the A in AH represents any anion that is a substrate of the anion exchanger.

Q_i	Amount of solute *i* per 10^{13} cells
$Q_\pm$	Net charge on impermeant cell ion (mEq(10^{13}cells)$^{-1}$))
C_i^c, C_j^m	Concentration of solute i in cell water (^{c}) or of solute j in extra-cellular medium (^{m}) (molar, M, for H^+ ions; millimolar, mM for all other solutes
Subscript i,j	All solutes or any subset of solutes among Na, K, Mg, A, Hb, X, Y, B, HB and H (*i*=*j* for all permeant solutes: Na, K, A, H)
Hb	Haemoglobin
A	Permeant anion
X	Global impermeant intracellular anion, assumed to be non-protonizable
$Y_\pm$	Impermeant extracellular monovalent ion (gluconate, N-methylglucamine or choline, for instance
B	Impermeant extracellular H^+ buffer (Hepes-like, in present simulation)
HB	Protonized form of extracellular H^+ buffer
K_B	Dissociation constant of extracellular H^+ buffer (M)
pH^c,pH^m	Cell and medium pH, respectively
n_x,n_{Hb}	Mean net charge on X and Hb, respectively (Eq(mol)$^{-1}$).
pI	Isoelectric pH of Hb
a	Slope of the proton titration curve of Hb (Eq(mol)$^{-1}$)[3,4]
f_{Hb}	Osmotic coefficient of Hb (osmol(mol)$^{-1}$)
b,c	Virial coefficients of linear and quadratic terms, in f_{Hb} equation [5]
z_i, F, R, T, E_m	Valence of ion *i*, Faraday constant, gas constant, absolute temperature and membrane potential (mV), respectively
E_i	Equilibrium potential of ion i (mV)
I_i	Electrodiffusional current across cell membrane (µA(10^{13}cells)$^{-1}$)
Φ_i	Total flux of permeant solute i (mmol(10^{13}cells)$^{-1}$))
$Y^{Hb}(t)$	Hb consumption
Y_{max}^{Hb}	Maximal Hb consumption (relative to initial Hb content of IRBC)
$t_{1/2}^{NPP}$	Half-time of NPP induction curve (*h*)
$t_{1/2}^{Hb}$	Half-time of Hb consumption curve (*h*)
s^{Hb}	Slope-parameter of the Hb-digestion curve (*h*)
s^{NPP}	Slope-parameter of the NPP induction curve (*h*)
cf	Coupling factor, determines parasite volume growth as fraction of cumulative cytostomal ingestions
p,q	Scaling factors of integration interval
V_W	Volume of cell water (l(10^{13}cells)$^{-1}$))
V_P	Parasite Volume at time t
V_P^0	Initial ring-stage parasite volume, assumed to occupy 4% of IRBC volume
V_H	Host cell volume at time t
V_H^0	Initial host cell volume normalized to 1
V_{IRBC}	Volume of infected cell at time t, relative to V_H^0

Partial flux component of solute i (mmol(10^{13}cells)$^{-1}$)h^{-1})

Φ_i^P	The Na^+ pump
Φ_i^G	Electrodiffusional constant-field channels (G)
Φ_i^{HA}	H:A (HA) cotransport (represents the Jacobs-Stewart mechanism, [1])
Φ_i^{KA}	K:Cl (KA) cotransport
Φ_i^{NaA}	Na:Cl (NaA) cotransport
Φ_i^{NaH}	Na:H (NaH) countertransport
$\Phi_{Na}^{F_{max}}, \Phi_{Na}^{R_{max}}$	Saturated forward (F) and reverse (R) Na^+ fluxes through the Na^+ pump
Φ_i^{NPP}	Flux of ion *i* through *P. falciparum*-induced new permeation pathway (NPP)
P_i^G	Permeability constant of solute *i* through electrodiffusional pathways (h^{-1})
$P_i^{NPP}(t)$	Time dependant permeability for ion *i* through the NPP
$P_{i,max}^{NPP}$	Maximum permeability for the ion *i* through the NPP (h^{-1})
k_{HA}	Rate constants for the H:A (HA) cotransport
k_{KA}	Rate constant of K:A cotransport
k_{NaA}	Rate constant for Na:A cotransport
Φ_W	water flux across cell membrane (l(10^{13}cells)$^{-1}$ h^{-1}))
D*t*	Integration interval
DV_W	Hb consumption-induced change in the volume of cell water per unit original volume of cells during one integration interval (l(10^{13}cells)$^{-1}$))
D*V'*	Homeostasis-induced change in host cell volume per unit original volume of cells during one integration interval (l(10^{13}cells)$^{-1}$))
DQ_i	Change in the intracellular amount of solute *i* during one integration interval (mmol(10^{13}cells)$^{-1}$))
r_A,r_H	Ratio between external and internal permeant anion concentrations (r_A) or between internal and external hydrogen ion concentrations (r_H)

Superscripts (*o*,*t* and *t*–D*t* on a variable indicate the value of that variable in the original reference steady state (*o*), at any subsequent time (*t*), or at the preceding integration interval (*t*–D*t*). The absence of a time-indicating superscript on any variable is equivalent to the superscript *t*. t_n is the n-th iteration in the numerical computation such that $t_{n+1}=t_n+$D*t*

Model equations

Reference State isotonicity

$$f_{Hb} \cdot C_{Hb}^{c} + C_{Na}^{c} + C_{K}^{c} + C_{Mg}^{c} + C_{X}^{c} + C_{A}^{c} = C_{Na}^{m} + C_{A}^{m} + C_{B}^{m} + C_{Y}^{m} \quad (1)$$

Reference State electroneutrality

$$Q_{Na}^{0} + Q_{K}^{0} + Q_{Mg}^{0} - Q_{A}^{0} - Q_{-}^{0} = 0 \quad (2)$$

$$C_{HB}^{m} = B \cdot \frac{C_{H}^{m}}{K_{B} + C_{H}^{m}} \quad (3)$$

H^{+} buffer behavior of Hb [3–5]

$$n_{Hb} = a(pH^{c} - pI) \quad (4)$$

$$Q_{-} = n_{Hb} \cdot Q_{Hb} + n_{X} \cdot Q_{X} + 2Q_{Mg} \quad (5)$$

Nonideal osmotic behavior of Hb [3–5]

$$f_{Hb} = 1 + b \cdot \frac{Q_{Hb}}{V_{W}} + c \cdot \left(\frac{Q_{Hb}}{V_{W}}\right)^{2} \quad (6)$$

Flux equations

Na^{+} pump-mediated fluxes (dissociation constant values from reference [6])

Forwards:

$$\Phi_{\mathrm{Na}}^{\mathrm{PF}} = -\Phi_{\mathrm{Na}}^{\mathrm{F_{max}}} \left(\frac{\mathrm{C_{Na}^{C}}}{\mathrm{C_{Na}^{C}} + 0.2(1 + \mathrm{C_{K}^{C}}/8.3)}\right)^{3} \left(\frac{\mathrm{C_{K}^{m}}}{\mathrm{C_{K}^{m}} + 0.1(1 + \mathrm{C_{Na}^{m}}/18)}\right)^{2} \quad (7)$$

Reverse:

$$\Phi_{\mathrm{Na}}^{\mathrm{PR}} = \Phi_{\mathrm{Na}}^{\mathrm{R_{max}}} \left(\frac{\mathrm{C_{K}^{C}}}{\mathrm{C_{K}^{C}} + 8.3(1 + \mathrm{C_{Na}^{C}}/0.2)}\right)^{2} \left(\frac{\mathrm{C_{Na}^{m}}}{\mathrm{C_{Na}^{m}} + 18(1 + \mathrm{C_{K}^{m}}/0.1)}\right)^{3} \quad (8)$$

Net:

$$\Phi_{\mathrm{Na}}^{\mathrm{P}} = \Phi_{\mathrm{Na}}^{\mathrm{PF}} + \Phi_{\mathrm{Na}}^{\mathrm{PR}} \quad (9)$$

$$\Phi_{\mathrm{K}}^{\mathrm{P}} = -\frac{\Phi_{\mathrm{Na}}^{\mathrm{P}}}{1.5} \quad (10)$$

Carrier-mediated cotransport (low-saturation kinetics)

$$\Phi_{Na,A}^{NaA} = -k_{NaA}(C_{Na}^{c} C_{A}^{c} - C_{Na}^{m} C_{A}^{m}) \quad (11\ A)$$

$$\Phi_{K,A}^{KA} = -k_{KA}(C_{K}^{c} C_{A}^{c} - C_{K}^{m} C_{A}^{m}) \quad (11\ B)$$

Jacobs-Stewart flux

$$\Phi_{\mathrm{H}}^{\mathrm{HA}} = \Phi_{\mathrm{A}}^{\mathrm{HA}} = -\mathrm{k}_{\mathrm{HA}}(\mathrm{C}_{\mathrm{A}}^{\mathrm{c}}\mathrm{C}_{\mathrm{H}}^{\mathrm{c}} - \mathrm{C}_{\mathrm{A}}^{\mathrm{m}}\mathrm{C}_{\mathrm{H}}^{\mathrm{m}}) \tag{12}$$

Electrodiffusional fluxes, including NPP-mediated fluxes

$$\Phi_{\mathrm{i}}^{\mathrm{G}} = -\mathrm{P}_{\mathrm{i}}^{\mathrm{G}} \cdot \left(\frac{z_i F E_m}{RT}\right) \cdot \left[\mathrm{C}_{\mathrm{i}}^{\mathrm{c}} - \mathrm{C}_{\mathrm{i}}^{\mathrm{m}} \cdot \exp\left(-\frac{z_i F E_m}{RT}\right)\right] \cdot (1 - \exp\left(-\frac{z_i F E_m}{RT}\right))^{-1} \tag{13}$$

$$\Phi_{\mathrm{Na}} = \Phi_{\mathrm{Na}}^{\mathrm{P}} + \Phi_{\mathrm{Na}}^{\mathrm{G}} + \Phi_{\mathrm{A}}^{\mathrm{NaA}} + \Phi_{\mathrm{Na}}^{\mathrm{NPP}} \tag{14}$$

$$\Phi_{\mathrm{K}} = \Phi_{\mathrm{K}}^{\mathrm{P}} + \Phi_{\mathrm{K}}^{\mathrm{G}} + \Phi_{\mathrm{K}}^{\mathrm{KA}} + \Phi_{\mathrm{K}}^{\mathrm{NPP}} \tag{15}$$

$$\Phi_{\mathrm{A}} = \Phi_{\mathrm{A}}^{\mathrm{G}} + \Phi_{\mathrm{A}}^{\mathrm{NaA}} + \Phi_{\mathrm{A}}^{\mathrm{KA}} + \Phi_{\mathrm{A}}^{\mathrm{HA}} + \Phi_{\mathrm{A}}^{\mathrm{NPP}} \tag{16}$$

$$\mathit{\Phi}_A = \mathit{\Phi}_A^G + \mathit{\Phi}_A^{HA} + \mathit{\Phi}_A^{NPP} \tag{17}$$

$$\mathit{\Phi}_W = P_W \cdot \left[f_{Hb} \cdot Q_{Hb} + \sum_i C_i^c - \sum_j C_j^m\right] \tag{18}$$

$$I_i = z_i \cdot F \cdot \Phi_{\mathrm{i}} \tag{19}$$

Maintenance of electroneutrality (neglecting capacitative transients)

$$\sum_i I_i = 0 \tag{20}$$

Time-dependent NPP permeability

$$P_i^{NPP,G}(t) = P_{i,max}^{NPP,G} \cdot \left(1 + \exp\left[\frac{t_{1/2}^{NPP} - t}{s^{NPP}}\right]\right)^{-1} \tag{21}$$

Hb consumption function

$$Y^{Hb}(t) = Y_{max}^{Hb} \cdot (1 + \exp\,[\frac{t_{1/2}^{Hb} - t}{s^{Hb}}])^{-1} \tag{22}$$

$$\Phi_{\mathrm{i}} = \frac{\mathrm{DQ_i}}{\mathrm{Dt}} \tag{23}$$

$$\mathrm{D}Q_i = \mathit{\Phi}_i \cdot \mathrm{D}t \tag{24}$$

$$Q_i^t = Q_i^{t-Dt} + \mathrm{D}Q_i \tag{25}$$

$$\mathrm{D}V_W = V_W^t - V_W^{(t-\mathrm{D}t)} \tag{26}$$

Host cytosol volume ingestion function:

$$V_W(t_{n+1}) = V_W(t_n) \cdot \frac{1 - Y^{Hb}(t_{n+1})}{1 - Y^{Hb}(t_n)} \tag{27}$$

Cumulative parasite volume growth

$$V_P = V_P^0 + cf \cdot \sum_{t_n} \mathrm{D}V_W \tag{28}$$

$$C_i^{c,t} = \frac{Q_i^t}{V_W^t} \quad (29)$$

Host cell volume at $t=t_n$

$$V_H = V_H^0 + \sum_{t_n} \mathrm{D}V' - \sum_{t_n} \mathrm{D}V_W \quad (30)$$

$$\mathrm{D}V' = \Phi_W \cdot \mathrm{D}t \quad (31)$$

$$\mathrm{D}t = \frac{p}{q+\sum|\Phi_i|} \quad (32)$$

$$pH^{c,m} = -\log C^{c,m} \quad (33)$$

$$r_A = \frac{C_A^m}{C_A^c} \quad (34)$$

$$r_H = \frac{C_H^c}{C_H^m} = \exp_{10}(pH^m - pH^c) \quad (35)$$

References

1. Lew VL, Bookchin RM (1986) Volume, pH and-ion content regulation in human red cells: analysis of transient behavior with an integrated model. J Membrane Biol 92: 57-74.

2. Lew VL, Freeman CJ, Ortiz OE, Bookchin RM (1991) A mathematical model on the volume, pH and ion content regulation of reticulocytes. Application to the pathophysiology of sickle cell dehydration. J Clin Invest 87: 100-112.

3. Cass A, Dalmark M (1973) Equilibrium dialysis of ions in nystatin-treated cells. Nature New Biol 244: 47-49.

4. Dalmark M (1975) Chloride and water distribution in human red cells. J Physiol 250: 65-84.

5. Freedman JC, Hoffman JF (1979) Ionic and osmotic equilibria of human red blood cells treated with nystatin. J Gen Physiol 74: 157-185.
